# Subsets of follicular lymphoma 3B have divergent outcomes: results from the prospective multicenter MER and LEO cohorts

**DOI:** 10.1038/s41408-025-01347-0

**Published:** 2025-08-08

**Authors:** Patrizia Mondello, Brianna Negaard, Andrew L. Feldman, Brian K. Link, Carla Casulo, Dai Chihara, David Russler-Germain, Jason Romancik, Caitlin Gribbin, Sara Haddadi, Eric Mou, Ivana N. Micallef, Patrick B. Johnston, Joseph Novak, Yucai Wang, Rebecca L. King, Anne J. Novak, Thomas M. Habermann, Peter Martin, Brad Kahl, Grzegorz S. Nowakowski, Loretta J. Nastoupil, James R. Cerhan, Christopher R. Flowers, Izidore S. Lossos, Richard W. Burack, Matthew J. Maurer, Stephen M. Ansell

**Affiliations:** 1 Division of Hematology, Mayo Clinic, Rochester, MN USA; 2 Department of Quantitative Health Sciences, Mayo Clinic, Rochester, MN USA; 3 Division of Hematopathology, Mayo Clinic, Rochester, MN USA; 4 Division of Hematology, Oncology and Bone Marrow Transplantation, University of Iowa, Iowa City, IA USA; 5 Wilmot Cancer Institute, University of Rochester, Rochester, NY USA; 6 MD Anderson Cancer Center, Houston, TX USA; 7 Washington University School of Medicine in St Louis, St Louis, MO USA; 8 Department of Hematology and Medical Oncology, Winship Cancer Institute at Emory University, Atlanta, GA USA; 9 Weill Cornell Medical College, New York, NY USA; 10 Division of Hematology, Sylvester Comprehensive Cancer Center, University of Miami, Miami, FL USA

**Keywords:** B-cell lymphoma, Chemotherapy

## Abstract

Follicular lymphoma (FL) 3B is considered an aggressive lymphoma, however recent studies have challenged this paradigm. Additional controversy involves the clinical implication of pure FL3B (FL3Bp) vs FL3B with concurrent diffuse large B cell lymphoma (DLBCL) (FL3Bc). To address these questions, we performed a pooled study of the MER and LEO cohorts comparing 464 newly diagnosed, R-CHOP-treated patients with FL1-2 (*n* = 216), FL3A (*n* = 170), FL3B (*n* = 78) and 739 DLBCL. Among FL3B patients, 19 (24%) had FL3Bc and 59 (76%) FL3Bp. Baseline characteristics and outcomes were similar between the two FL3B subtypes. Compared to FL1-3A, FL3B showed similar clinical features, except for a lower tumor burden. After R-CHOP, FL1-2 patients had an inferior event-free survival (EFS) than those with FL3B, whereas there was no difference with FL3A. Survival was similar across the FL grades. Although FL1-2 patients failed to achieve EFS24 more frequently than FL3B and FL3A, FL3B patients who failed EFS24 had three-fold higher risk of subsequent mortality than other FLs. At 5-year follow-up FL3B patients had twice the risk of relapse with an aggressive subtype than those with FL1-2 and FL3A. Compared to DLBCL, FL3B patients had more favorable clinical features, but similar outcomes to GCB subtype. Our data suggest that most FL3B have a good outcome, while a subset has an aggressive behavior.

## Introduction

Follicular lymphoma (FL) is the most common indolent lymphoma with a generally favorable outcome [1]. In the revised 4^th^ edition of the World Health Organization (WHO) classification, FL is graded from 1 to 3 based on the number of centroblasts per high powered field. FL grade 3 is further subdivided into FL3A and FL3B, depending on the presence or absence of centrocytes, respectively [2]. However, histological grading is a matter of debate due to poor reproducibility and subjectivity of counting centroblasts, and similar outcomes between patients with FL1-2 and FL3A [3–7]. While the 5^th^ edition of the WHO classification of hematolymphoid tumors (WHO-HAEM5) does not mandate grading and instead combines FL1-2 and FL3A in a unique entity termed classic FL (cFL), the International Consensus Classification (ICC) still recommends grading [8]. Nevertheless, both classifications set apart FL3B, which appears to behave more aggressively, although the WHO-HAEM5 has renamed this entity follicular large B cell lymphoma.

Among FL grades, FL3B is the least common, accounting for 5–10% of FL cases [9]. Almost half of FL3B tumors have concurrent diffuse large B cell lymphoma (DLBCL) in the diagnostic tissue biopsy [5, 10] However, as FL3B and DLBCL share the same cytomorphology, the only difference between them is the histopathologic growth pattern which is follicular in FL3B and diffuse in DLBCL [2]. Moreover, pure FL3B shares the same genetic mutations of FL3B with DLBCL, thus they may represent a pathologic continuum rather than transformation of disease, in contrast to low-grade FL [10–12]. Notably, FL3B rarely co-exists with FL1-2 or FL3A or harbors *BCL2* rearrangements, suggesting a different pathogenesis [13–15].

Several lines of evidence suggest that FL3B displays distinct biological and clinical characteristics resembling aggressive DLBCL rather than indolent, lower-grade FL1-2 and FL3A [10, 13, 15, 16]. Accordingly, DLBCL anthracycline-containing treatment regimens, such as R-CHOP, are currently the mainstay of therapy for FL3B with or without co-existing DLBCL. While some studies show that R-CHOP therapy failed to induce remissions in 40% of FL3B patients and was associated with a higher mortality than those with FL1-2 and FL3A with 5-year overall survival (OS) rate of 54% [17, 18], others report outcomes comparable to FL3A with 5-year OS of 79–90% [5, 6, 19]. These conflicting results are likely due to differences in studied populations, the retrospective nature of investigation, lack of rigorous pathology review, the rarity of the disease and variability in treatment. Controversial results have also emerged regarding the clinical impact of FL3B with or without co-existing DLBCL [10, 20, 21]. Koch et al. did not find differences in clinical presentation and outcomes between patients with pure FL3B vs those with concurrent DLBCL [10], while other studies observed a worse prognosis in the latter group [20, 21]. Therefore, it remains unclear whether FL3B displays aggressive clinical behavior with an inferior survival or has a higher risk to relapse as DLBCL or high-grade B-cell lymphoma (HGBCL). It is similarly debatable whether concurrence of FL3B with DLBCL (FL3Bc) rather than a pure FL3B (FL3Bp) is associated with a worse presentation and prognosis. To address these open questions, we used two large prospective multicenter study cohorts to compare the baseline characteristics and outcomes of newly diagnosed FL3B with FL1-2, FL3A and DLBCL patients, all of whom were uniformly treated with frontline R-CHOP.

## Methods

### Patients

Patients were prospectively enrolled in the Iowa/Mayo SPORE Molecular Epidemiology Resource (MER) [22] and the Lymphoma Epidemiology of Outcomes (LEO) [23] multicenter cohorts between 2002 and 2021. Seventy-eight patients with FL3B (either FL3Bp or FL3Bc) treated with frontline R-CHOP regimen were compared to patients with FL1-2 (*n* = 216) and FL3A (*n* = 170) who received R-CHOP within 6 months of diagnosis based on the physician’s choice, and de novo DLBCL (*n* = 739) without underlying FL or associated FL3B. All patients were 18 years or older. The clinical stage of the disease was determined based on the medical history and physical examination; complete blood count; serum biochemical profile; computed tomography (CT) scan of the chest, abdomen and pelvis; positron emission tomography (PET) scan with FDG whenever available; and bone marrow core biopsy of the iliac crest as per standard institutional guidelines. All patients were systematically followed for disease progression/relapse, re-treatment and death; all events were validated against medical records.

### Ethics approval and consent to participate

The patients signed written informed consent to participate in the MER and LEO cohorts. This study was reviewed and approved by the Mayo Clinic Institutional Review Board and the Institutional ethics committees of all participating LEO centers. The study was conducted according to the Declaration of Helsinki.

### Histological evaluation

FL3B cases were identified in clinical files and designated as FL3Bc and FL3Bp based on the presence or absence of co-existing DLBCL, respectively. Similarly, FL1-2 and FL3A were based on the original diagnosis. DLBCL was classified as germinal center B cell (GCB) and non-GCB according to the Hans algorithm based on immunohistochemistry (IHC). FL3B with co-existing FL1-2 or FL3A, or composite DLBCL with lower-grade FL were excluded. FL unclassifiable cases were also excluded in this study to ensure stringent analysis. Histologic diagnosis was determined according to the WHO 4^th^ edition criteria until 2016 [24] and revised 4^th^ edition criteria [22] thereafter by an expert pathologist at each participating center, and all cases were reviewed by a designated pathologist at each MER and LEO site to provide the final diagnosis.

### Molecular classification

We used existing RNA-sequencing (RNA-seq) and whole exome sequencing (WES) data generated at Mayo Clinic [25] to classify 275 DLBCL based on DZsign [26]. Classification method and tools for analysis were used as previously described [25, 26].

### Statistical analysis

Event free survival (EFS) was defined as time from diagnosis to progression, relapse, retreatment, or death. Early progression was defined as failing to achieve EFS at 24 months (EFS24) [27, 28] OS was defined as time from diagnosis to death from any cause. Due to shorter follow-up of the LEO cohort, all the outcomes were truncated at 5 years. Associations between clinical features and EFS or OS were evaluated using Kaplan–Meier curves and Cox proportional hazards models unadjusted and adjusted for age and FLIPI (comparisons with FL1-2 and FL3A) or IPI (comparisons with DLBCL). EFS24 was compared using logistic regression; subtype at relapse was evaluated using cumulative incidence with competing risks. Comparisons of clinical features between subgroups was performed using two-sample *t*-test, ANOVA tests and chi-squared tests as appropriate. All *p*-values are reported unadjusted for multiple comparisons and 95% confidence intervals are reported. A *p*-value < 0.05 was considered significant. Analysis was performed using R v4.2.2.

## Results

### Patient characteristics

Seventy-eight patients with FL3B, 386 with FL1-3A (FL1-2, *n* = 216 and FL3A, *n* = 170) and 739 with DLBCL (GCB DLBCL, *n* = 455 and non-GCB DLBCL, *n* = 284) who were treated with frontline R-CHOP regimen within 6 months of diagnosis were prospectively enrolled into the SPORE MER and LEO cohorts from 2002 to 2021. Median age was 61 years (range, 18–94 years) and 56% were male. After censoring, the median follow-up for the analysis was 60 months. Characteristics of the pooled cohorts are reported in Supplementary Table 1.

#### FL3B patients with and without concurrent DLBCL have similar clinical features and outcomes

**Fig. 1:**
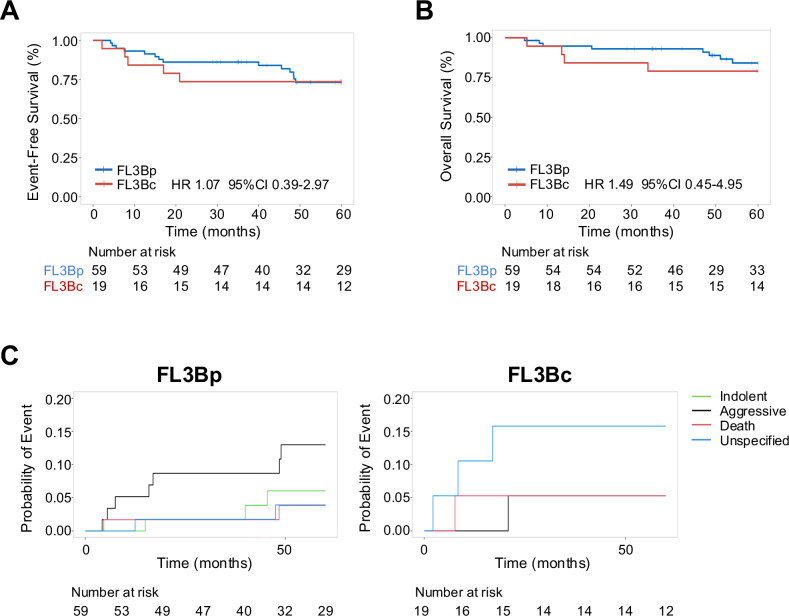
FL3B with or without co-existing DLBCL are clinically similar. Kaplan–Meier curves representing event-free survival (**A**) and overall survival (**B**) in patients with pure FL3B (FL3Bp, blue) and FL3B with concurrent DLBCL (FL3Bc, red). **C** Cumulative incidence of relapse with indolent (green), aggressive (black) or unspecified (light blue) disease, and death (red) by FL3B subtype.

Initially, we investigated whether having FL3Bc vs FL3Bp makes a difference in clinical presentation and outcome. Among FL3B patients, 19 (24%) had FL3Bc and 59 (76%) FL3Bp histology. A diagnostic excisional biopsy was obtained in 15 (79%) and 46 (78%; *p* = 0.928) patients, respectively. Baseline characteristics were broadly similar between these two FL3B subtypes, except for a higher frequency of bone marrow involvement (18% vs 0%; *p* < 0.001) in FL3Bp (Supplementary Table 2). There was no difference in EFS (unadjusted HR 1.07 95% CI 0.39–2.97, adjusted HR 1.12 95% CI 0.36–3.49; Fig. 1A) and OS (unadjusted HR 1.49 95% CI 0.45–4.95, adjusted HR 1.94 95% CI 0.46–8.1 Fig. 1B) between the two groups. Although more patients with FL3Bc failed EFS24 compared to those with FL3Bp (26% vs 14%; Supplementary Table 3), there was no difference in subsequent outcome (unadjusted HR 1.27 95% CI 0.35–4.58, adjusted HR 1.19 95% CI 0.16–8.71; Supplementary Fig. 1).

Next, we investigated whether the nature of relapses differed between the two FL3B groups (Supplementary Table 3). At 2-year follow-up, there was a higher rate of biopsy-proven aggressive relapses (namely DLBCL or HGBCL) in FL3Bp compared to FL3Bc patients (9% vs 5%), and this persisted over time (13% in FL3Bp vs 5% in FL3Bc at 5 years). Few patients with FL3Bp had an indolent histology (namely FL1-2 and FL3A) at first relapse (2% at 2 year and 6% at 5 year), while this was not observed in FL3Bc patients. Interestingly, at 2 years there were more unspecified relapses, defined as requiring medical attention even though not biopsied, in patients with FL3Bc compared to FL3Bp (16% vs 2%), and this difference remained at 5 years (16% vs 4%; Fig. 1C). However, the number of patients was small to achieve a conclusion. Altogether, acknowledging the risk for type two error, these data suggest that patients with FL3Bc present with sufficiently similar clinical features and survival outcomes to those with FL3Bp. This clinical observation combined with the similar genomics of FL3B with or without DLBCL [10] justify combining FL3B histologic subtypes for subsequent analysis.

#### A subset of FL3B patients relapse early and have a worse outcome than FL1-2 and FL3A patients

**Fig. 2:**
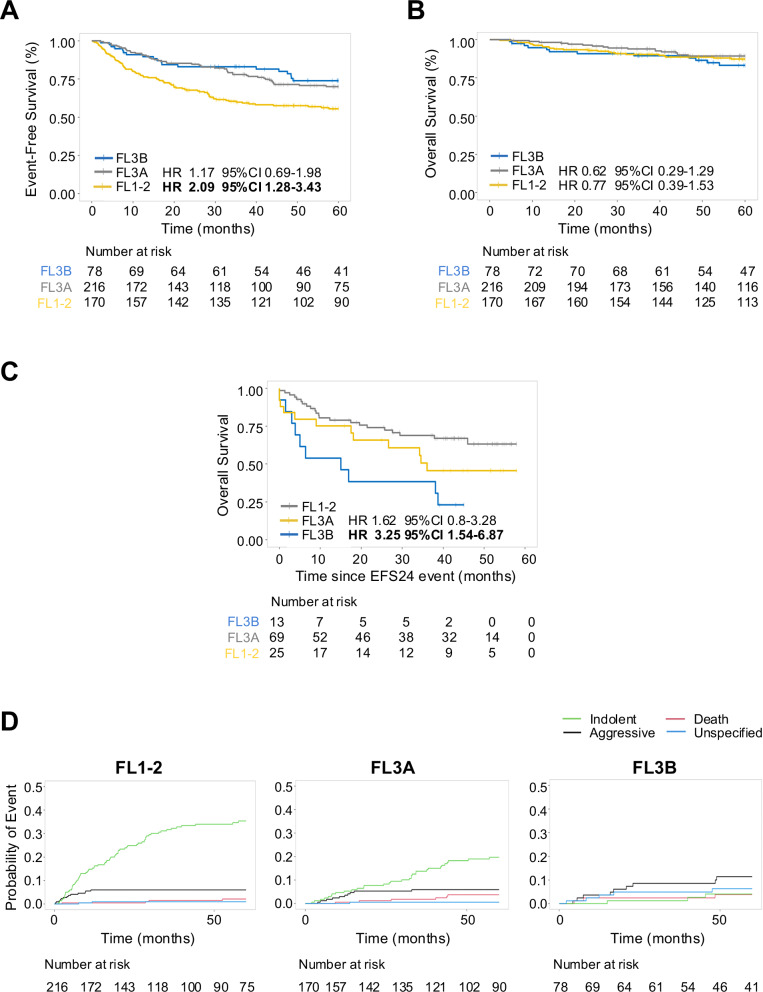
A subset of FL3B patients relapse early and have a worse outcome than those with lower-grade FL. Kaplan–Meier curves representing event-free survival (**A**) and overall survival (**B**) in patients with FL3B (blue), FL1-2 (yellow) and FL3A (gray). **C** Kaplan–Meier curves representing overall survival from the time of failing EFS24 in patients with FL3B (blue), FL1-2 (yellow) and FL3A (gray). **D** Cumulative incidence of relapse with indolent (green), aggressive (black) or unspecified (light blue) disease, and death (red) in FL1-2, FL3A and FL3B patients.

We then compared all FL3B patients to those with lower-grade FL. Notably, patients with FL3B less frequently had advanced stage disease (III-IV) (*p* = 0.008), >4 nodal groups involved (*p* < 0.028), or bone marrow involvement (*p* = 0.002) than those with FL1-2 and FL3A. Moreover, FL3B patients had a higher frequency of elevated serum LDH values (*p* = 0.002) and anemia (*p* = 0.003) than those with FL3A, but similar to FL1-2 (Table 1). Compared to FL3B, patients with FL1-2 had an inferior EFS (unadjusted HR 2.09 95% CI 1.28–3.43, adjusted HR 1.96 95% CI 1.19–3.26) whereas no difference was observed with those with FL3A (unadjusted HR 1.17, 95% CI 0.69–1.98, adjusted HR 1.20, 95% CI 0.7–2.05; Fig. 2A). However, there was no survival disadvantage in the FL1-2 cohort (unadjusted HR 0.77 95% CI 0.39–1.53, adjusted HR 0.69 95% CI 0.35–1.39; Fig. 2B) compared to other FL groups.

**Table 1:** Patient characteristics of FL1-2, FL3A, FL3B.

	FL1-2 (*n* = 216)	FL3A (*n* = 170)	FL3B (*n* = 78)	*p*-value
Age at diagnosis				0.258
Mean (SD)	58 (12)	59 (14)	60 (13)	
Range	27–85	22–86	27–94	
Age > 60	92 (43%)	84 (49%)	38 (49%)	0.362
Sex				0.346
Female	86 (40%)	80 (47%)	35 (45%)	
Male	130 (60%)	90 (53%)	43 (55%)	
LDH abnormal	83 (45%)	41 (27%)	28 (41%)	0.002
BM involvement	79 (37%)	47 (28%)	13 (17%)	0.002
Hb < 12 g/dL	44 (23%)	19 (13%)	22 (31%)	0.003
Missing data	24	21	8	
Stage				0.008
I-II	43 (20%)	50 (30%)	28 (36%)	
III-IV	171 (80%)	115 (70%)	49 (64%)	
Missing data	2	5	1	
Nodal groups > 4	98 (48%)	63 (39%)	23 (31%)	0.028
Extranodal group > 1	39 (19%)	26 (16%)	7 (9%)	0.171
FLIPI				0.197
0–1	60 (33%)	67 (42%)	29 (46%)	
2	59 (32%)	49 (31%)	20 (32%)	
3–5	63 (34%)	43 (27%)	14 (22%)	

*FL* follicular lymphoma, *SD* standard deviation, *LDH* lactate dehydrogenase, *BM* bone marrow, *Hb* hemoglobin, *FLIPI* follicular lymphoma international prognostic index.

The EFS24 failure rate was comparable in patients with FL3B and FL3A (17% and 15%), while it was higher in patients with FL1-2 (34%, *p* < 0.001; OR 2.47 95% CI 1.31–4.99) (Supplementary Table 4). As expected, the FL3B patients who failed to achieve EFS24 had a worse prognosis compared to those who achieved EFS24 (HR 36.64 95% CI 8.61–182.43; Supplementary Fig. 2A). A similar observation was found in the FL1-2 (HR 18.13 95% CI 5.41–60.68; Supplementary Fig. 2B) and FL3A groups (HR 21.24 95% CI 7.44–60.63; Supplementary Fig. 2C). Notably, compared to FL1-2, FL3B who failed EFS24 had three times higher risk of subsequent mortality (HR 3.25 95% CI 1.54–6.87), while there was no significant difference compared to FL3A (HR 1.62 95% CI 0.8–3.28) (Fig. 2C).

Although patients with FL1-2 relapsed more often than those with other FLs, there was a substantial difference in the nature of first relapse (Supplementary Table 4). At 5 years of follow-up, FL3B patients had almost twice the risk of relapse with an aggressive subtype (DLBCL or HGBCL) (*n* = 8, 11%) compared to those with FL1-2 (*n* = 13, 6%) and FL3A (*n* = 10, 6%; *p* < 0.001), suggesting a distinct underlying biology. In contrast, patients with FL1-2 and FL3A more frequently recurred with indolent disease (FL1-2 and FL3A) (*n* = 72, 33% and *n* = 32, 19%) than those with FL3B (*n* = 3, 4%) (Fig. 2D). Collectively, these data identify a small subset of FL3B patients (17%) who relapse early, frequently with an aggressive histology and have a worse prognosis than early progressors with FL1-2 and FL3A. However, the majority of FL3B patients show similar outcome to the FL1-2 and FL3A groups treated with R-CHOP.

#### FL3B patients have more favorable clinical features than those with DLBCL, but similar outcome to GCB subtype

To clarify whether FL3B resembles DLBCL in clinical features and outcomes, we compared the patients with FL3B to those with DLBCL. The baseline characteristics of patients differed significantly for lower rate of extranodal involvement (*p* = 0.002) in the FL3B group, which however more often had bone marrow involvement (*p* < 0.001) and >4 nodal groups involved (*p* < 0.001) than those with DLBCL (Supplementary Table 5). Compared to FL3B, patients with DLBCL had a trend toward lower EFS (unadjusted HR 1.50 95% CI 0.94–2.4, adjusted HR 1.26 95% CI 0.79–2.01; Supplementary Fig. 3A) and OS (unadjusted HR 1.70 95% CI 0.95–3.04, adjusted HR 1.41 95% CI 0.79–2.53; Supplementary Fig. 3B). The risk to fail EFS24 was higher in DLBCL compared to FL3B patients (OR 1.85 95% CI 1.04–3.62; Supplementary Table 6), but with no difference in subsequent survival between the two groups (unadjusted HR 1.20 95% CI 0.63–2.27, adjusted HR 1.41 95% CI 0.73–2.74; Supplementary Fig. 3C) likely due to power limitation.

**Fig. 3:**
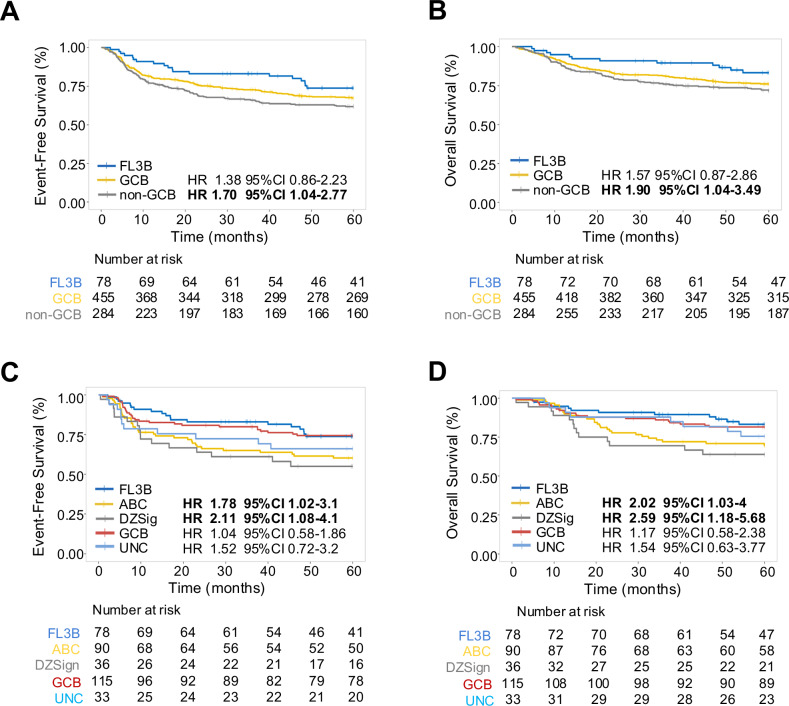
FL3B patients have similar outcomes than those with GCB DLBCL. Kaplan–Meier curves representing event-free survival (**A**) and overall survival (**B**) in patients with FL3B (blue), GCB DLBCL (yellow) and non-GCB DLBCL (gray). Kaplan–Meier curves representing event-free survival (**C**) and overall survival (**D**) in patients with FL3B (blue), ABC (yellow), DZsig (gray), GCB (red) and Unclassified (UNC) (light blue) DLBCL.

To further investigate similarity between FL3B and distinct DLBCL subtypes, we distinguished DLBCL based on cell-of-origin (COO) in GCB and non-GCB types. At diagnosis, patients with FL3B differed significantly from those with GCB and non-GCB DLBCL for lower frequency of abnormal LDH (*p* = 0.049), anemia (*p* < 0.001) and extranodal disease (*p* = 0.008), while they more often had >4 nodal groups (*p* < 0.001; Table 2). Compared to FL3B, patients with non-GCB DLBCL had an inferior EFS (unadjusted HR 1.70 95% CI 1.04–2.77, adjusted HR 1.35 95% CI 0.83–2.21; Fig. 3A) and OS (unadjusted HR 1.90 95% CI 1.04–3.49, adjusted HR 1.49 95% CI 0.81–2.74; Fig. 3B), while those with GCB DLBCL did not show a significant survival difference (EFS unadjusted HR 1.38 95% CI 0.86–2.23, adjusted HR 1.20 95% CI 0.74–1.94; OS unadjusted HR 1.57, 95% CI 0.87–2.86, adjusted HR 1.36, 95% CI 0.75–2.47). Patients with FL3B failed EFS24 less frequently than patients with GCB or non-GCB DLBCL (17% vs 25% and 33%, *p* = 0.016; Supplementary Table 7). Among patients who failed EFS24, those with FL3B had a similar subsequent OS as both GCB (unadjusted HR 1.25 95% CI 0.65–2.41, adjusted HR 1.61 95% CI 0.81–3.18) and non-GCB DLBCL (unadjusted HR 1.13 95% CI 0.58–2.19, adjusted HR 1.21 95% CI 0.61–2.42; Supplementary Fig. 4).

**Table 2:** Patient characteristics of FL3B and DLBCL by COO.

	FL3B (*n* = 78)	GCB DLBCL (*n* = 455)	Non-GCB DLBCL (*n* = 284)	*p*-value
Age at diagnosis				0.413
Mean (SD)	60 (13)	62 (15)	63 (14)	
Range	27–94	18–90	19–93	
Age > 60	38 (49%)	273 (60%)	166 (58%)	0.175
Sex				0.365
Female	35 (45%)	210 (46%)	116 (41%)	
Male	43 (55%)	245 (54%)	168 (59%)	
LDH abnormal	28 (41%)	211 (50%)	148 (56%)	0.049
BM involvement	13 (17%)	50 (11%)	46 (16%)	<0.001
Hb < 12 g/dL	22 (31%)	120 (28%)	122 (46%)	<0.001
Missing data	8	30	20	
Stage				0.175
I-II	28 (36%)	194 (43%)	103 (36%)	
III-IV	49 (64%)	260 (57%)	181 (64%)	
Missing data	1	1	0	
Nodal groups > 4	23 (31%)	24 (5%)	18 (6%)	<0.001
Extranodal group > 1	7 (9%)	114 (25%)	72 (25%)	0.008
IPI				0.052
0–1	38 (49%)	175 (39%)	86 (30%)	
2	20 (26%)	113 (25%)	79 (28%)	
3	15 (19%)	110 (24%)	76 (27%)	
4–5	5 (6%)	57 (13%)	43 (15%)	

*FL* follicular lymphoma, *GCB* germinal center B cell, *DLBCL* diffuse large B cell lymphoma, *COO* cell of origin, *SD* standard deviation, *LDH* lactate dehydrogenase, *BM* bone marrow, *Hb* hemoglobin, *IPI* international prognostic index.

Finally, to explore whether FL3B may underline the biology of high-risk GCB patients, we reclassified our DLBCL cohort based on DZsig [26]. Baseline characteristics of patients with FL3B differed from those with GCB and DZsig for a younger age (*p* = 0.046), a higher rate of bone marrow involvement (*p* < 0.001) and >4 nodal groups (*p* = 0.015; Supplementary Table 8). Compared to FL3B, patients with ABC and DZsig had an inferior EFS (ABC: unadjusted HR 1.78 95% CI 1.02–3.1, adjusted HR 1.45 95% CI 0.82–2.57; DZsig: unadjusted HR 2.11 95% CI 1.08–4.1, adjusted HR 1.73 95% CI 0.88–3.39) and OS (ABC: unadjusted HR 2.02 95% CI 1.03–4, adjusted HR 1.45 95% CI 0.73–2.91; DZsig: unadjusted HR 2.59 95% CI 1.18–5.68, adjusted HR 1.96 95% CI 0.89–4.33), while those with GCB and unclassified (UNC) had comparable survival (Fig. 3C, D). The risk to fail EFS24 was also higher in patients with ABC (OR 2.38 95% CI 1.15–5.16) and DZsig (OR 2.70 95% CI 1.09–6.74) than those with FL3B, while it was similar to GCB (OR 1.15 95% CI 0.55–2.51) and UNC (OR 1.87 95% CI 0.69–4.93). Together these data suggest that patients with FL3B have more favorable clinical presentation and outcome compared to DLBCL. When subdividing DLBCL based on COO, FL3B patients have a similar prognosis than those with GCB subtype, although this finding would need to be evaluated further in a larger study.

## Discussion

Here we present the first prospective multicenter study comparing FL3B patients to those with FL1-2, FL3A, and DLBCL uniformly treated with the frontline R-CHOP regimen. Our analyses find that FL3B with or without DLBCL have similar clinical features and outcomes. We also show that most patients with FL3B have a prognosis comparable to FL1-2 and FL3A. However, 17% of FL3B patients relapse within 24 months from diagnosis, frequently with an aggressive histology and an inferior survival than early progressors with FL1-2 and FL3A. Finally, we report that FL3B patients have better clinical features and prognosis than those with overall DLBCL, even though they have similar prognosis to GCB subtype.

Over the last two decades, the aggressive nature of FL3B has been a topic of debate, as it remains unclear whether having concordant DLBCL in the diagnostic biopsy makes a difference in outcome. In our analysis, patients with FL3Bp displayed similar clinical and prognostic features to those with co-existing DLBCL. This observed lack of difference might be secondary to insufficient statistical power. Furthermore, a coexistent DLBCL component could be missed even with excisional biopsy, as assessment of one anatomic site may not necessarily capture disease heterogeneity at other sites. However, since FL3B with and without concurrent DLBCL have similar genetic alterations [10], it is also possible that they may represent a disease continuum of FL progressing towards DLBCL as supported by their different growth pattern (follicular vs diffuse) and nature of relapse (indolent vs aggressive). Our data are in line with Koch et al. who also reported that patients with pure FL3B have similar clinical presentation and outcomes to those with coexisting DLBCL [10]. These findings apparently contrast with two prior studies identifying more aggressive behavior in FL3B with concurrent DLBCL [20, 21], but this discordance may be secondary to differing populations and case selection, lack of rigorous pathology review and/or a limited statistical power.

When compared to FL1-2 and FL3A, FL3B showed similar clinical characteristics, except for a lower tumor burden. This observation is likely influenced by the selection bias of more adverse clinical features (including extensive disease) required to initiate intensive R-CHOP treatment in patients with lower-grade FL. As in prior studies [5, 19, 29], we observed a similar EFS in FL3B and FL3A patients, whereas recurrences were more frequent in FL1-2 cases. However, there was no difference in survival across the FL grades. Although this observation is in contrast with prior studies reporting a higher mortality in FL3B [17, 18, 21]. it is possible that the diverse therapeutic approaches, sometimes not including rituximab, and the smaller number of cases represent confounders of older investigations.

Despite a higher rate of recurrences in FL1-2, we found that the nature of relapses differed substantially across the FL groups. FL3B had a higher incidence of an aggressive subtype at first relapse, as opposed to the indolent relapses (especially lower grade FL) observed in FL1-2 and FL3A. This different pattern may be secondary to the selection of one or more resistant clones under chemotherapy pressure [30–32], or it might be linked to genetic and epigenetic alterations [10, 33–35] associated with FL3B biology. Alternatively, the slow-growing cells in FL1-2 and FL3A emerge because inherently more resistant to chemotherapy than those that proliferate rapidly, whereas FL3B recurs as large cells due to its large cell nature. Future studies integrating complementary platforms, especially at the single-cell level, are warranted to deconvolute the complex dynamic and heterogeneity of intratumoral clonal subpopulations of FL3B and decode how the interaction between the tumor clones and the tumor microenvironment may favor malignant transformation.

Early progression has long been identified as a robust predictor of reduced survival [36]. While this endpoint has been validated in several studies [27, 37–40], it is not clear whether all FL grades with early progression have poor outcomes. In our study, failing to achieve EFS24 was confirmed to impact survival. However, this negative effect was worse in FL3B patients compared with those with FL1-2 and FL3A, suggesting that the molecular framework underlying FL3B matters.

Additional discordance occurs with some studies reporting that FL3B patients have clinical features and prognosis like DLBCL [17, 18], while others pointing towards similarity to lower-grade FL [5, 6, 19]. To address this open question, we further compared FL3B patients to a cohort of DLBCL. Notably, patients with FL3B had more favorable clinical features and outcomes to those with DLBCL. However, after subdividing DLBCL based on COO, FL3B had a similar prognosis as GCB-subtype. Collectively, our data suggest that FL3B is a unique entity within the lymphoma spectrum and exhibits clinical features intermediate between FL1-2/3A and DLBCL. This concept is supported by three gene expression studies that revealed a distinct biologic profile of FL3B compared to lower grade FL and DLBCL [10, 41, 42]. Piccaluga et al. found that FL1-2 and FL3A clustered together, while FL3B formed a separated distinguishable cluster, even though this group was more similar to FL1-3A than to DLBCL [41]. Horn et al. observed that FL3B was similar to FL3A, and both formed an intermediate cluster between FL1-2 and DLBCL [42]. Koch et al. showed that pure FL3B and FL3B with simultaneous DLBCL share similar genetic and transcriptional alterations, which in turn diverge from de novo DLBCL [10]. Future studies should therefore identify the cell-of-origin of FL3B and clarify the biologic interconnection with lower-grade FL and DLBCL.

From a therapeutic perspective, identification of the progenitor from which FL3B arises would be of paramount importance. It is possible that the subset of FL3B with early progression has a distinct cell-of-origin and molecular profiles which dictate more aggressive clinical features and predict decrease response to therapy. Future attention should be also directed to the tumor microenvironment which may play a role in promoting and/or corroborating tumor growth and survival as in other lymphomas [28, 35, 43]. Therefore, identification of markers that allow stratification of patients for personalized approaches, development of prognostic biomarkers, and identification of targets for therapy would be critical in this unfavorable category of lymphoma patients.

Despite being the largest prospective cohort, the main limitation of this study is the relatively small number of patients with FL3B, given the rarity of this diagnosis. An additional limitation is the limited IHC and FISH diagnostic data available, which precluded more detailed analysis. The strengths of this study are the central pathology review, the uniform treatment in a multicenter setting and the relative long follow-up with annotated histology of relapses.

## Conclusions

In this prospective multicenter study, 17% of FL3Bs relapse early, often with an aggressive subtype, and have a poor prognosis, thus providing consideration for different therapeutic approaches. In contrast, the majority of FL3B have more favorable clinical characteristics and outcome. Future studies are warranted to identify the distinct molecular and immunologic mechanisms that drive FL3B and that can be targeted therapeutically.

## Supplementary information

Supplemental Material

## Data Availability

Deidentified data from this study will be made available on request to the corresponding authors.
