# Supplementary material for: Subsets of follicular lymphoma 3B have divergent outcomes: results from the prospective multicenter MER and LEO cohorts
Source: Blood Cancer J. 2025 Aug 8;15(1):134. doi: 10.1038/s41408-025-01347-0 (PMC12334739; doi:10.1038/s41408-025-01347-0)
Supplement: Supplementary file 1 — Supplemental Material [file 41408_2025_1347_MOESM1_ESM.doc]

Mondello P, et al., Supplementary Materials

**Subsets of follicular lymphoma 3B have divergent outcomes: results from the prospective multicenter MER and LEO cohorts**

Contents

**SUPPLEMENTARY TABLES**

**Supplementary Table 1**. Characteristics of the study cohort.

**Supplementary Table 2**. Patient characteristics of FL3B overall and classified based on concurrent DLBCL or pure disease features.

**Supplementary Table 3**. Relapse events of FL3B overall and classified based on concurrent DLBCL or pure disease features

**Supplementary Table 4**. Relapse events of FL1-2, FL3A and FL3B.

**Supplementary Table 5**. Patient characteristics of FL3B and DLBCL.

**Supplementary Table 6**. Relapse events of FL3B and DLBCL.

**Supplementary Table 7**. Relapse events of FL3B and DLBCL by COO.

**Supplementary Table 8.** Patient characteristics of FL3B and DLBCL by DZ signature.

**SUPPLEMENTARY FIGURES**

**Supplementary Figure 1**: FL3B with or without concurrent DLBCL are clinically similar.

**Supplementary Figure 2**: FL3B patients who failed EFS24 have an inferior subsequent survival than those with FL1-2 and FL3A.

**Supplementary Figure 3**: FL3B patients have more favorable than those with DLBCL.

**Supplementary Figure 4**: FL3B patients who failed EFS24 have a similar subsequent survival than those with GCB and non-GCB DLBCL.

**Supplementary Table 1.** Characteristics of the study cohort.

|  | **FL1-2**  (n=216) | **FL3A**  (n=170) | **FL3B**  (n=78) | **DLBCL**  (n=739) | **p-value** |
| --- | --- | --- | --- | --- | --- |
| **Age at diagnosis**  Mean (SD)  Range | 58 (12)  27-85 | 59 (14)  22-86 | 60 (12)  27-94 | 62 (14)  18-93 | <0.001 |
| **Sex**  Female  Male | 86 (40%)  130 (60%) | 80 (47%)  90 (53%) | 35 (45%)  43 (55%) | 326 (44%)  413 (56%) | 0.533 |
| **EFS status**  Event  No Event | 91 (42%)  125 (58%) | 7 (33%)  14 (67%) | 19 (24%)  59 (76%) | 250 (34%)  489 (66%) | 0.002 |
| **EFS24**  Achieved  Failed  Missing | 133 (66%)  69 (34%)  14 | 142(85%)  25 (15%)  3 | 62 (83%)  13 (17%)  3 | 511 (72%)  200 (28%)  28 | <0.001 |
| **OS status**  Alive  Deceased | 191 (88%)  25 (12%) | 153 (90%)  17 (10%) | 71 (85%)  12 (15%) | 553 (75%)  186 (25%) | <0.001 |

**Abbreviations:** FL, follicular lymphoma; GCB, germinal center B cell; DLBCL, diffuse large B cell lymphoma; SD, standard deviation; FU, follow-up; mo, months; OS, overall survival; EFS, event free survival, N/A, not applicable.

.

**Supplementary Table 2.** Patient characteristics of FL3B overall or classified based on concurrent DLBCL or pure disease features.

|  | **FL3B**  **(n=78)** | **FL3Bp**  **(n=59)** | **FL3Bc**  **(n=19)** | **p-value** |
| --- | --- | --- | --- | --- |
| **Age at diagnosis**  Mean (SD)  Range | 60 (12)  27-94 | 61 (13)  27-94 | 59 (9)  40-72 | 0.574 |
| **Age >60** | 38 (49%) | 29 (49%) | 9 (47%) | 0.892 |
| **Sex**  Female  Male | 35 (45%)  43 (55%) | 27 (46%)  32 (54%) | 8 (42%)  11 (58%) | 0.780 |
| **LDH abnormal** | 28 (41%) | 20 (40%) | 8 (44%) | 0.743 |
| **BM involvement** | 13 (14%) | 11 (18%) | 0 (0%) | <0.001 |
| **Hb < 12 g/dL**  Missing data | 22 (31%)  8 | 17 (33%)  7 | 5 (28%)  1 | 0.699 |
| **Stage**  I-II  III-IV  Missing data | 28 (36%)  49 (64%)  1 | 22 (38%)  36 (62%)  1 | 6 (32%)  13 (68%)  0 | 0.617 |
| **Nodal groups >4** | 23 (31%) | 20 (36%) | 3 (17%) | 0.129 |
| **Extranodal group >1** | 7 (9%) | 5 (9%) | 2 (10%) | 0.619 |
| **FLIPI**  0-1  2  3-5  Missing data | 29 (46%)  20 (32%)  14 (22%)  15 | 23 (50%)  15 (33%)  8 (17%)  13 | 6 (35%)  5 (29%)  6 (36%)  2 | 0.299 |
| **IHC markers**  **CD10**  Positive  Negative  Missing data  **CD5**  Positive  Negative  Missing data  **MUM1**  Positive  Negative  Missing data  **BCL2**  Positive  Negative  Missing data  **BCL6**  Positive  Negative  Missing data  **CD20**  Positive  Negative  Missing data  **KI67**  <=20  >20 <=50  >50  Missing data | 37 (71%)  15 (29%)  26  4 (14%)  25 (86%)  49  13 (52%)  12 (48%)  53  55 (90%)  6 (10%)  17  46 (100%)  0 (0%)  32  54 (100%)  -  24  4 (10%)  11 (27%)  26 (63%)  37 | 31 (67%)  15 (33%)  13  4 (15%)  22 (85%)  33  12 (54%)  10 (46%)  37  40 (89%)  5 (11%)  14  39 (100%)  0 (0%)  20  47 (100%)  -  12  4 (11%)  10 (26%)  24 (63%)  21 | 6 (100%)  0 (0%)  16  3 (100%)  0 (0%)  16  1 (34%)  2 (66%)  16  15 (94%)  1 (6%)  3  7 (100%)  0 (0%)  12  7 (100%)  0 (0%)  12  0 (0%)  1 (33%)  2 (67%)  16 | N/A  N/A  N/A  N/A  N/A  N/A  N/A |
| **Bone marrow infiltration**  Involved by large cells  Involved by small cells/FL  Negative  Missing data | 3 (11%)  2 (7%)  23 (82%)  50 | 2 (7%)  3 (7%)  23 (86%)  32 | 1 (100%)  0 (0%)  0 (0%)  18 | N/A |

**Abbreviations:** FL, follicular lymphoma; FL3Bp, pure follicular lymphoma 3B; FL3Bc, follicular lymphoma 3B with concurrent diffuse large B cell lymphoma; SD, standard deviation; LDH, lactate dehydrogenase; BM, bone marrow; Hb, hemoglobin; FLIPI, follicular lymphoma international prognostic index; IHC, immunohistochemistry; N/A, non-applicable as formal statistical analysis was not performed due to the large amount of missing data.

**Supplementary Table 3.** Relapse events of FL3B overall and classified based on concurrent DLBCL or pure disease features.

|  | **FL3B**  **(n=78)** | **FL3Bp**  **(n=59)** | **FL3Bc**  **(n=19)** | **p-value** |
| --- | --- | --- | --- | --- |
| **EFS24**  Achieved  Failed  Missing data | 62 (83%)  13 (17%)  3 | 48 (86%)  8 (14%)  3 | 14 (74%)  5 (26%)  0 | 0.231 |
| **EFS**  Event  No event | 19 (24%)  59 (76%) | 14 (24%)  45 (76%) | 5 (26%)  14 (74%) | 0.819 |
| **First Event**  Aggressive  Death  Indolent  Unspecified  None | 8 (10%)  3 (4%)  3 (4%)  5 (6%)  63 (76%) | 7 (12%)  2 (3%)  3 (5%)  2 (3%)  45 (77%) | 1 (5%)  1 (5%)  0 (0%)  3 (16%)  14 (74%) | 0.271 |
| **FU**  Alive  Deceased | 66 (85%)  12 (15%) | 51 (86%)  8 (14%) | 15 (79%)  4 (21%) | 0.431 |

**Abbreviations:** FL, follicular lymphoma; FL3Bp, pure follicular lymphoma 3B; FL3Bc, follicular lymphoma 3B with concurrent diffuse large B cell lymphoma; EFS, event free survival; FU, follow-up.

**Supplementary Table 4.** Relapse events of FL1-2, FL3A and FL3B.

|  | **FL 1-2**  **(n=216)** | **FL3A**  **(n=170)** | **FL3B**  **(n=78)** | **p-value** |
| --- | --- | --- | --- | --- |
| **EFS24**  Achieved  Failed  Missing data | 133 (66%)  69 (34%)  14 | 142 (85%)  25 (15%)  3 | 62 (83%)  13 (17%)  3 | <0.001 |
| **EFS**  Event  No event | 91 (42%)  125 (58%) | 49 (42%)  125 (58%) | 19 (24%)  59 (76%) | 0.003 |
| **First Event**  Aggressive  Death  Indolent  Unspecified  None | 13 (6%)  4 (2%)  72 (33%)  2 (1%)  121 (71%) | 10 (6%)  6 (3%)  32 (19%)  1 (1%)  121 (71%) | 8 (10%)  3 (4%)  3 (4%)  5 (6%)  59 (76%) | <0.001 |
| **FU**  Alive  Deceased | 191 (88%)  25 (12%) | 153 (90%)  17 (10%) | 66 (85%)  12 (15%) | 0.470 |

**Abbreviations:** FL, follicular lymphoma; EFS, event free survival; FU, follow-up.

**Supplementary Table 5.** Patient characteristics of FL3B and DLBCL.

|  | **FL3B**  (n=78) | **DLBCL**  (n=739) | **p-value** |
| --- | --- | --- | --- |
| **Age at diagnosis**  Mean (SD)  Range | 60 (13)  27-94 | 62 (14)  18-93 | 0.286 |
| **Age >60** | 38 (49%) | 439 (59%) | 0.069 |
| **Sex**  Female  Male | 35 (45%)  43 (55%) | 326 (41%)  413 (59%) | 0.898 |
| **LDH abnormal** | 28 (41%) | 359 (52%) | 0.079 |
| **BM involvement** | 13 (17%) | 96 (13%) | <0.001 |
| **Hb < 12 g/dL**  Missing data | 22 (31%)  8 | 242 (35%)  30 | 0.536 |
| **Stage**  I-II  III-IV  Missing data | 28 (36%)  49 (64%)  1 | 297 (40%)  441 (60%)  1 | 0.508 |
| **Nodal groups >4** | 23 (31%) | 42 (13%) | <0.001 |
| **Extranodal group >1** | 7 (9%) | 186 (25%) | 0.002 |
| **IPI**  0-1  2  3  4-5 | 38 (49%)  20 (26%)  15 (19%)  5 (6%) | 261 (35%)  192 (26%)  186 (25%)  100 (14%) | 0.064 |

**Abbreviations:** FL, follicular lymphoma; GCB, germinal center B cell; DLBCL, diffuse large B cell lymphoma; COO, cell of origin; SD, standard deviation; LDH, lactate dehydrogenase; BM, bone marrow; Hb, hemoglobin; IPI, international prognostic index.

**Supplementary Table 6.** Relapse events of FL3B and DLBCL.

|  | **FL3B**  **(n=78)** | **DLBCL**  **(n=739)** | **p-value** |
| --- | --- | --- | --- |
| **EFS24**  Achieved  Failed  Missing data | 62 (83%)  13 (17%)  3 | 511 (72%)  200 (28%)  28 | 0.045 |
| **EFS**  Event  No event | 19 (24%)  59 (76%) | 250 (34%)  489 (66%) | 0.091 |
| **First Event**  Aggressive  Death  Indolent  Unspecified  None | 8 (10%)  3 (4%)  3 (4%)  5 (6%)  59 (76%) | N/A  67 (9%)  183 (25%)  489 (66%)  0 (0%) | <0.001 |
| **FU**  Alive  Deceased | 66 (85%)  12 (15%) | 553 (75%)  186 (25%) | 0.055 |

**Abbreviations:** FL, follicular lymphoma; EFS, event free survival; FU, follow-up.

**Supplementary Table 7.** Relapse events of FL3B and DLBCL by COO.

|  | **FL3B**  **(n=78)** | **GCB DLBCL**  (n=455) | **Non-GCB DLBCL**  (n=284) | **p-value** |
| --- | --- | --- | --- | --- |
| **EFS24**  Achieved  Failed  Missing data | 62 (83%)  13 (17%)  3 | 326 (75%)  111 (25%)  18 | 185 (72%)  89 (28%)  10 | 0.016 |
| **EFS**  Event  No event | 19 (24%)  59 (76%) | 144 (32%)  311 (68%) | 106 (37%)  178 (63%) | 0.067 |
| **First Event**  Aggressive  Death  Indolent  Unspecified  None | 8 (10%)  3 (4%)  3 (4%)  5 (6%)  59 (76%) | N/A  38 (8%)  106 (23%)  311 (68%)  0 (0%) | N/A  29 (10%)  77 (27%)  178 (63%)  0 (0%) | <0.001 |
| **FU**  Alive  Deceased | 66 (85%)  12 (15%) | 144 (32%)  311 (68%) | 106 (38%)  178 (62%) | 0.066 |

**Abbreviations:** FL, follicular lymphoma; EFS, event free survival; FU, follow-up.

**Supplementary Table 8.** Patient characteristics of FL3B and DLBCL by DZ signature.

|  | **FL3B**  (n=78) | **GCB**  (n=115) | **ABC**  (n=90) | **DZsig**  (n=36) | **UNC**  (n=33) | **p-value** |
| --- | --- | --- | --- | --- | --- | --- |
| **Age at diagnosis**  Mean (SD)  Range | 60 (13)  27-94 | 62 (15)  18-90 | 66 (11)  39-89 | 64 (13)  24-85 | 64 (13)  31-87 | 0.125 |
| **Age >60** | 38 (49%) | 71 (62%) | 61 (68%) | 27 (75%) | 20 (61%) | 0.046 |
| **Sex**  Female  Male | 35 (45%)  43 (55%) | 52 (45%)  63 (55%) | 36 (40%)  54 (60%) | 19 (53%)  17 (47%) | 10 (30%)  23 (70%) | 0.375 |
| **LDH abnormal** | 28 (41%) | 45 (43%) | 46 (54%) | 16 (49%) | 17 (47%) | 0.442 |
| **BM involvement** | 13 (17%) | 8 (7%) | 15 (16%) | 3 (8%) | 6 (18%) | <0.001 |
| **Hb < 12 g/dL**  Missing data | 22 (31%)  8 | 26 (25%)  11 | 37 (43%)  4 | 8 (25%)  4 | 11 (34%)  1 | 0.094 |
| **Stage**  I-II  III-IV  Missing data | 28 (36%)  49 (64%)  1 | 57 (50%)  58 (50%)  0 | 35 (39%)  55 (61%)  0 | 14 (39%)  22 (61%)  0 | 17 (52%)  16 (48%)  0 | 0.263 |
| **Nodal groups >4** | 23 (31%) | 5 (9%) | 8 (24%) | 1 (7%) | 2 (14%) | 0.015 |
| **Extranodal group >1** | 7 (9%) | 27 (24%) | 20 (23%) | 9 (25%) | 7 (21%) | 0.131 |
| **IPI**  0-1  2  3  4-5 | 38 (49%)  20 (26%)  15 (19%)  5 (6%) | 175 (39%)  113 (25%)  110 (24%)  57 (13%) | 48 (42%)  30 (26%)  28 (24%)  9 (8%) | 12 (33%)  9 (25%)  11 (31%)  4 (11%) | 12 (36%)  9 (27%)  7 (21%)  5 (15%) | 0.259 |

**Abbreviations:** FL, follicular lymphoma; GCB, germinal center B cell; DLBCL, diffuse large B cell lymphoma; DZsig, dark zone signature; UNC, unclassified; SD, standard deviation; LDH, lactate dehydrogenase; BM, bone marrow; Hb, hemoglobin; IPI, international prognostic index.

**Supplementary Figure 1.** Kaplan-Meier curves representing overall survival from the time of failing

EFS24 in patients with pure FL3B (FL3Bp, blue) and FL3B with concurrent DLBCL (FL3Bc, red).

**Supplementary Figure 2. A-C.** Kaplan-Meier curves representing overall survival in patients who achieved (blue) or failed (red) EFS24 with FL3B (**A**), FL1-2 (**B**), and FL3A (**C**).

**Supplementary Figure 3. A-B.** Kaplan-Meier curves representing event-free survival (**A**) and overall survival (**B**) in patients with FL3B (blue) and DLBCL (yellow). **C.** Kaplan-Meier curves representing overall survival from the time of failing EFS24 in patients with FL3B (blue) and DLBCL (yellow).

**Supplementary Figure 4. A.** Kaplan-Meier curves representing overall survival from the time of failing EFS24 in patients with FL3B (blue), GCB DLBCL (yellow) and non-GCB DLBCL (gray).
